# Patient perceptions about laparoscopy at Komfo Anokye Teaching Hospital, Ghana

**DOI:** 10.11604/pamj.2015.20.422.6218

**Published:** 2015-04-29

**Authors:** Adam Gyedu, Setri Fugar, Raymond Price, Juliane Bingener

**Affiliations:** 1 Department of Surgery, Kwame Nkrumah University of Science and Technology, Kumasi, Ghana; 2 Directorate of Surgery, Komfo Anokye Teaching Hospital, P. O. Box 1934, Kumasi, Ghana; 3 Department of Surgery, University of Utah, Salt Lake City, USA; 4 Department of Surgery, Intermountain Medical Center, Intermountain Healthcare, Salt Lake City, USA; 5 Mayo Clinic, Rochester, Minnesota, USA

**Keywords:** Patient perception, Laparoscopy, Ghana

## Abstract

**Introduction:**

Laparoscopy has become the gold standard for many surgical cases in the developed world. It however, remains a rarity in developing countries for several reasons, a major one being cost. This study aimed to determine the knowledge and attitude of patients attending Komfo Anokye Teaching Hospital (KATH) in Kumasi, Ghana toward laparoscopic surgery and their willingness to pay for it.

**Methods:**

A cross-sectional survey was conducted among patients attending specialist clinics at KATH.

**Results:**

1070 patients participated. Mean age was 40±15years. 54% were city-dwellers. 14% had salary-paying jobs. None had undergone prior laparoscopic surgery. 3% had knowledge of laparoscopy. 95% preferred laparoscopy to open surgery mainly because of faster recovery and less post-op pain. Age >45years (AOR = 0.53, p = 0.03) and higher education (AOR = 2.00, p = 0.04) were significant predictors of patient choice. Among those preferring laparoscopy, 78% were willing to pay more than the baseline cost of open surgery for laparoscopy. A history of previous abdominal surgery (AOR = 0.67, p = 0.02), having a salaried job compared with being unemployed (AOR = 2.36, p < 0.01) and living in the city compared with the village (AOR = 1.78, p = 0.04) were significant predictors of patients’ willingness to pay more for laparoscopy.

**Conclusion:**

Knowledge about laparoscopy and its benefits are severely lacking among patients at KATH. Once educated about its benefits, most people prefer laparoscopy even if they needed to pay more for it even in resource-limited countries like Ghana.

## Introduction

Minimally invasive surgery (MIS) revolutionized the surgical world 20 years ago and changed the approach of surgical procedures forever. Currently, MIS procedures are the standard of care for many operations such as laparoscopic appendectomy and cholecystectomy in developed countries [1]. However, laparoscopic surgery and other forms of minimally invasive surgery are not routinely found in resource-restricted countries including Ghana. The reasons include increased operative cost of minimally invasive procedures as well as the lack of local training opportunities in laparoscopic techniques [2]. Some successful laparoscopic programs have been established in resource-restricted countries [3–5]. The advancement of MIS has largely been a patient-driven process. The rapid introduction and spread of laparoscopic cholecystectomy in the developed world was driven mostly by public demand as the benefits of decreased pain and suffering became known [6]. Reports in the English literature, that evaluate the knowledge and attitudes of patients in resource-restricted countries about laparoscopy are lacking. This study aimed to assess the knowledge and attitudes of patients in Ghana toward laparoscopic surgery. The specific goals were to determine what percentage of patients knew about laparoscopic surgery, the proportion that would prefer laparoscopy to open surgery and the proportion of those choosing laparoscopy that would be willing to pay above the baseline cost of open surgery.

## Methods

A cross-sectional study was carried out between July and August 2013 at the surgical specialist outpatient clinics of Komfo Anokye Teaching Hospital (KATH), Ghana. The KATH serves most of the northern sector of Ghana and records an average of 123 visits to surgical specialist outpatient clinics per day. By assuming that 50% of the population knew about laparoscopy, with a 95% confidence interval (CI) and 6% margin of error, a sample size of 1 069 was calculated. The study population included all patients attending surgical specialist outpatient clinics. Data were collected on every weekday within the period. Participants were selected systematically based on how they were seated at the clinic. Starting from the first patient in the first row of seats every third patient was approached and after explaining the study and seeking consent, the questionnaire was administered through a face-to-face interview. The participants were interviewed using a structured questionnaire (see Appendix). To allow for effective quantification, majority of the questions posed were constructed with yes/no answers. Participants were asked if they knew about laparoscopic surgery. Those who knew were asked a set of questions to assess their knowledge. To those with no knowledge of laparoscopy, an explanation of the procedure was given including the benefits and drawbacks compared with open surgery. Photos of the incision scars following cholecystectomy performed by open and laparoscopic techniques were shown to them. Participants were then asked to choose between open and laparoscopic surgery if they needed a surgery and the reasons behind their choice. Multiple reasons were permitted. Those who chose laparoscopy were then asked how much extra they were willing to pay in addition to the cost of open surgery in order to have laparoscopic surgery. Demographics such as age, education level, employment status, location of residence and history of previous abdominal surgery were also collected. Data analysis was done with STATA V.11 (StataCorp, College Station, TX). The proportions of patients with knowledge of and preferences to laparoscopy were calculated. Secondary analysis compared proportions across demographics. Odds ratios (OR) and 95% confidence intervals were calculated. Adjusted ORs were calculated by multiple logistic regression methods to estimate the effect of various demographic variants (e.g age, education, place of residence and type of job) on participants’ preference of laparoscopy and also on their willingness to pay for it. Significant variables (p < 0.05) were included in the logistic model, the “best model” selected according to the Aikaike Information Criterion value.

## Results

Table 1 summarizes the characteristics of the participants. One thousand and seventy patients participated in the study. The mean age was 40±15 years. Fifty-four percent lived in the city while 38% lived in a town [7]. Sixty percent of them had completed basic education (nine years of education including junior high school) or less. The majority (86%) were either engaged in hourly-paying jobs (farmers, artisans, food vendors, etc) or were unemployed. Twenty-three percent had a history of prior abdominal surgery and none had a history of previous laparoscopic surgery. Only 31(3%) participants knew about laparoscopic surgery, with 29/31 correctly identifying characteristics of laparoscopic surgery. After further education about laparoscopy and open surgery, when presented with a choice, 1015 (95%) patients preferred laparoscopy instead of open surgery most commonly because of faster recovery and less post-op pain (Table 2). Multivariate analysis (Table 3) showed that older patients (>45 years) were 47% less likely to prefer laparoscopic surgery compared with younger patients (≤45 years) (p = 0.03). Also, patients with educational status of senior high or higher were twice more likely to choose laparoscopy compared with those with a lower educational status (p = 0.04). Among those who preferred laparoscopy, 793(78%) were willing to pay some amount above the baseline cost of open surgery for laparoscopy. Out of the 793; 479 (60%) were willing to pay GHS800-1000 (400-500 US$) more for laparoscopy (Table 4). Average annual household income in Ghana is about GHS1 217 (1327 US$) whilst the average per capita income is almost GHS400 (433 US$) [8]. Multivariate analysis (Table 5) showed that patients living in the city were more likely to be willing to pay extra for laparoscopy compared with those living in the village (Adjusted Odds Ratio (AOR) = 1.78, p = 0.04). Likewise, patients who had a salaried job were more likely to pay extra compared with those who were unemployed (AOR = 2.36, p < 0.01). Patients who had a history of previous abdominal surgery were less likely to be willing to pay extra for laparoscopy compared with those who had no history of previous abdominal surgery (AOR = 0.67, p = 0.02). For the 55 (5%) participants who preferred open surgery to laparoscopy, the main factor that drove their choice was their familiarity with the open surgery (56%). Only 21% of those choosing open surgery stated financial factors as the reason behind their choice.

**Table 1:** Sample characteristics

Variable	Have knowledge about lap	Prefer lap to open surgery	Will pay for laparoscopy	Total
**n**	31	1 015	793	1 070
**Age (mean ± SD), years**	30±8	40±15	39±15	40±15
**Sex (%, 95% CI)**				
Female	45 (36-73)	53 (50-56)	51 (47-54)	53 (50-56)
Male	55 (27-64)	47 (44-46)	49 (46-53)	47 (45-50)
**Age ≤45 years (%, 95% CI)**	94 (84-100)	68 (65-70)	70 (66-73)	67 (64-69)
**Location**+ **(%, 95% CI)**				
Village	0.0 (0.0)	8.0 (6.2-9.5)	7.0 (5.3-8.9)	8.0 (6.4-9.7)
Town	3.0 (3.3-9.8)	38 (35-41)	36 (33-39)	38 (36-41)
City	97 (90-100)	54 (51-57)	57 (53-60)	54 (51-57)
**Senior high education or more (%, 95% CI)**	100 (100)	41 (38-44)	42 (39-46)	40 (37-43)
**Employment status (%, 95% CI)**				
Unemployed	26 (9.5-42)	27 (25-30)	26 (23-29)	27 (24-30)
Hourly worker	3.0 (0.0-9.8)	58 (55-61)	58 (55-62)	59 (56-62)
Salaried worker	71 (54-89)	14 (12-16)	16 (14-19)	14 (12-16)
**Previous surgery (%, 95% CI)**	23 (7.0-38)	27 (24-30)	26 (23-29)	28 (25-30)
**Previous abdominal surgery (%, 95% CI)**	19 (5.0-34)	22 (19-25)	21 (18-23)	23 (20-25)

+ Definition of location by population size: Village: 5 000 or less; Town: 5 000-250 000; City: 250 000 or more.

**Table 2:** Characteristics of respondents who prefer laparoscopy, N = 1 015

Reasons for preference	(%)
Faster recovery	36
Less postop pain	22
Better cosmesis	16
Shortened LOS+	10
Faster return to work	10
Less wound complication	1.9
Others++	5.6

+ LOS: length of hospital stay

++ Including better because more expensive, modern, better visibility, better expertise

**Table 3:** Predictors of patient preference for laparoscopy: univariate and multivariate regression models

	Univariate Model			Multivariate Model		
Patient Variables	Crude OR+	95% CI	P-value	Adjusted OR	(95% CI)	P-value
**Age**						
≥45 vs <45 (years)	**0.43**	**0.25, 0.74**	**0.002**	**0.53**	**0.30, 0.93**	**0.03**
**Sex**						
Female vs male	1.25	0.72, 2.15	0.42			
**Education status**						
≥Senior high vs <Senior high	**2.45**	**1.28, 4.70**	**0.007**	**2.00**	**0.33, 3.91**	**0.04**
**Employment status**						
Hourly worker vs Unemployed	0.69	0.35, 1.35	0.28			
Salaried worker vs Unemployed	1.04	0.38, 2.84	0.08			
**Location**						
Town vs village	1.20	0.47, 3.05	0.69			
City vs village	1.64	0.65, 4.13	0.29			
**Previous surgery**						
Yes vs no	**0.51**	**0.29, 0.89**	**0.02**	0.58	0.33, 1.03	0.06
**Previous abdominal surgery**						
Yes vs no	**0.53**	**0.30, 0.95**	**0.03**			

+ OR: odds ratio

**Table 4:** Additional amount+ patients who prefer and are willing to pay for laparoscopy (N = 1 015)

Amount (GHS/US$)++	%
None	22
400-600/200-300	31
800-1 000/400-500	47

+ Above the basic cost of open surgery

++ GHS: Ghana Cedis; US$: United States Dollars

**Table 5:** Predictors of patient willingness to pay for laparoscopy: Univariate and Multivariate Regression Models

	Univariate Model			Multivariate Model		
Patient Variables	Crude OR	(95% CI)	P-value	Adjusted OR	(95% CI)	P-value
**Age**						
≥45 vs <45 years	**0.68**	**0.50, 0.93**	**0.02**			
**Sex**						
Female vs male	**0.69**	**051, 0.94**	**0.02**			
**Education status**						
≥Senior high vs <Senior higher	**1.41**	**1.03, 1.93**	**0.03**			
**Employment Status**						
Hourly worker vs unemployed	1.22	0.87, 1.70	0.24	1.36	0.97, 1.91	0.08
Salaried worker vs Unemployed	**2.47**	**1.41, 4.33**	**0.002**	**2.36**	**1.34, 4.16**	**0.003**
**Location**						
Town vs village	1.14	0.67, 1.95	0.63	1.08	0.63, 1.86	0.77
City vs village	**1.85**	**1.09, 3.15**	**0.02**	**1.78**	**1.03, 3.05**	**0.04**
**Previous surgery**						
Yes vs no	0.78	0.57, 1.09	0.15			
**Previous abdominal surgery**						
Yes vs no	**0.70**	**0.50, 0.98**	**0.04**	**0.67**	**0.47, 0.95**	**0.02**

## Discussion

There are many studies in the literature that have focused on the feasibility of implementing laparoscopic procedures in resource-poor countries and how to overcome the challenges involved [9–11]. Literature focused on how patients in resource-poor countries perceive laparoscopy is however limited [12]. This study was done to assess the knowledge and perception of patients attending specialist outpatient clinics in KATH toward laparoscopic surgery. The 3% knowledge prevalence about laparoscopic surgery in the population was very low. This likely contributes greatly to the historically low demand for laparoscopy. However, the overwhelming majority of patients preferred laparoscopy to open surgery after the pros and cons of both procedures were explained to them. Their choice of laparoscopy was mainly influenced by its promise of faster recovery, less postop pain and better cosmetic outcome. Similar findings have been reported by other studies assessing patient choice between single incision versus multiport laparoscopic cholecystectomy and transvaginal versus transabdominal approach to the abdomen [13, 14]. Preference for laparoscopy was not influenced by patient's gender, location where they lived, their employment status or whether they had previous abdominal surgery. Patients of a higher educational status were at increased odds of choosing laparoscopy. Other studies have identified similar findings relating to the relationship of higher education and the increased desire for minimally invasive procedures [14, 15]. Older patients were less likely to prefer laparoscopy. Risk aversion and lack of enthusiasm to embrace a new procedure may contribute to this finding. It is informative that more than half of the patients preferring open surgery to laparoscopy said they preferred the open technique because it was more familiar to them. Seventy-eight percent of patients who chose laparoscopic surgery were willing to pay extra for the procedure. The majority of these patients were willing to pay up to 1000GHS (500 US$) above the baseline price for open surgery. While cost of surgery has been well documented as an impediment to the establishment of sustainable laparoscopic programs in resource-restricted countries, the data suggests that when the procedure is well explained to patients they will be willing to pay for it.

The data also suggests that salaried workers are 2.4 times more likely to pay for laparoscopy compared with the unemployed (p < 0.01). Hourly workers were only 40% more likely to pay for laparoscopy compared with the unemployed (p > 0.05). Since their income is based on the time spent at work, one would expect hourly workers to be more interested in faster postop recovery and earlier return to work and hence be more willing to pay for laparoscopy compared with salaried workers. There is a perceived low value of lost working-time in Ghana, as is the case in many resource-restricted countries, which significantly affects medical care. For many patients, immediate return to work may not be critical. Strong extended families allow for effective temporary relief of a patient's household duties and responsibilities. Therefore, a shorter hospital stay is often not sought. Many patients, in fact, frequently prefer (for transportation and cultural reasons) in-patient care to day surgery. Cost of in-patient care is also low [4, 12]. City dwellers were more likely to pay for laparoscopy. City dwellers were also better educated than town-dwellers and village-dwellers respectively (53% vs 28% vs 7%) and were more engaged in salaried jobs (19% vs 10% vs 2%). Contrary to expectations, patients who had previously undergone abdominal surgery were less likely to prefer laparoscopy. They were also less likely pay for it. The disinterest in laparoscopy in patients who previously had abdominal surgery, in fact, may be due to skepticism that minimally invasive procedures could actually accomplish such complex surgeries [15]. From the data, patients who had undergone previous abdominal surgery were 67% more likely to be older (>45 years) (p = 0.001). This older age group was less likely to prefer laparoscopy and also less likely to pay for it. There are several limitations of the study. Many of the questions restricted participants to yes/no answers. This could limit the open comment opportunity to elicit patients’ true preferences and to illustrate the sociocultural aspects of the study population [13]. We may also have introduced a bias towards patients’ preference for laparoscopy by giving detailed descriptions about the possible risks and benefits of laparoscopy, which are not well known by our patient population in comparison with the open approach. Despite these limitations, this study gives important insights into how patients in a resource-restricted country perceive laparoscopy.

## Conclusion

The overwhelming majority of patients attending specialist outpatient clinics in KATH do not know about laparoscopy. However, they overwhelmingly prefer laparoscopy if the option is made available to them. A high proportion of patients are also willing to pay for laparoscopy. Public education about the benefits of laparoscopy may lead to increased patient demand as occurred in developed countries. Health facilities wishing to provide patient centered care in Ghana should anticipate this increased demand and begin to develop the necessary human and physical resources through training and acquisition of the necessary equipment.

## References

[1] Bucher P, Pugin F, Ostermann S, Ris F, Chilcott M, Morel P (2011). Population perception of surgical safety and body image trauma: a plea for scarless surgery?. Surg Endosc..

[2] Hamamci EO, Besim H, Bostanoglu S, Sonisik M, Korkmaz A (2002). Use of laparoscopic splenectomy in developing countries: analysis of cost and strategies for reducing cost. J Laparoendosc Adv Surg Tech A..

[3] Adisa AO, Alatise OI, Arowolo OA, Lawal OO (2012). Laparoscopic appendectomy in a Nigerian teaching hospital. JSLS..

[4] Clegg-Lamptey JNA, Amponsah G (2010). Laparoscopic cholecystectomy at Korle-Bu Teaching Hospital, Accra, Ghana: An initial report. West Afri J Med..

[5] Vargas G, Price RR, Sergelen O, Lkhagvabayar B, Batcholuun P, Enkhamagalan T (2012). A successful model for laparoscopic training in mongolia. International surgery..

[6] Perissat J (1999). Laparoscopic surgery: A pioneer's point of view. World journal of surgery..

[7] Owusu G (2005). Small Towns in Ghana: Justifications for their Promotion under Ghana ‘s Decentralisation Programme. African Studies Quarterly..

[8] Ghana Statistical Service (2008). Ghana living standards survey: Report of the fifth round (GLSS 5).

[9] Adisa AO, Lawal OO, Alatise OI, Adesunkanmi ARK (2011). An Audit of Laparoscopic Surgeries in Ile-Ife, Nigeria. West Afr J Med..

[10] Okrainec A, Henao O, Azzie G (2010). Telesimulation: an effective method for teaching the fundamentals of laparoscopic surgery in resource-restricted countries. Surg Endosc..

[11] Straub CM, Price RR, Matthews D, Handrahan DL, Sergelen D (2011). Expanding laparoscopic cholecystectomy to rural Mongolia. World journal of surgery..

[12] Poenaru D, Steffes B Developing world perceptive on minimally invasive surgery and training, 2010. Minimally invasive surgery training: theories, methods, outcomes (Internet).

[13] Bingener J, Sloan JA, Ghosh K, McConico A, Mariani A (2012). Qualitative and quantitative analysis of women's perceptions of transvaginal surgery. Surg Endosc..

[14] Hey J, Roberts KJ, Morris-Stiff GJ, Toogood GJ (2012). Patient views through the keyhole: new perspectives on single-incision vs; multiport laparoscopic cholecystectomy. HPB (Oxford)..

[15] Swanstrom LL, Volckmann E, Hungness E, Soper NJ (2009). Patient attitudes and expectations regarding natural orifice translumenal endoscopic surgery. Surg Endosc..

